# Extraction of human kinase mutations from literature, databases and genotyping studies

**DOI:** 10.1186/1471-2105-10-S8-S1

**Published:** 2009-08-27

**Authors:** Martin Krallinger, Jose MG Izarzugaza, Carlos Rodriguez-Penagos, Alfonso Valencia

**Affiliations:** 1 Structural Biology and BioComputing Programme, Spanish National Cancer Research Centre, Madrid, Spain; 2 Barcelona Media, Centre d'Innovació, Av. Diagonal 177, Barcelona, Spain

## Abstract

**Background:**

There is a considerable interest in characterizing the biological role of specific protein residue substitutions through mutagenesis experiments. Additionally, recent efforts related to the detection of disease-associated SNPs motivated both the manual annotation, as well as the automatic extraction, of naturally occurring sequence variations from the literature, especially for protein families that play a significant role in signaling processes such as kinases. Systematic integration and comparison of kinase mutation information from multiple sources, covering literature, manual annotation databases and large-scale experiments can result in a more comprehensive view of functional, structural and disease associated aspects of protein sequence variants. Previously published mutation extraction approaches did not sufficiently distinguish between two fundamentally different variation origin categories, namely natural occurring and induced mutations generated through in vitro experiments.

**Results:**

We present a literature mining pipeline for the automatic extraction and disambiguation of single-point mutation mentions from both abstracts as well as full text articles, followed by a sequence validation check to link mutations to their corresponding kinase protein sequences. Each mutation is scored according to whether it corresponds to an induced mutation or a natural sequence variant. We were able to provide direct literature links for a considerable fraction of previously annotated kinase mutations, enabling thus more efficient interpretation of their biological characterization and experimental context. In order to test the capabilities of the presented pipeline, the mutations in the protein kinase domain of the kinase family were analyzed. Using our literature extraction system, we were able to recover a total of 643 mutations-protein associations from PubMed abstracts and 6,970 from a large collection of full text articles. When compared to state-of-the-art annotation databases and high throughput genotyping studies, the mutation mentions extracted from the literature overlap to a good extent with the existing knowledgebases, whereas the remaining mentions suggest new mutation records that were not previously annotated in the databases.

**Conclusion:**

Using the proposed residue disambiguation and classification approach, we were able to differentiate between natural variant and mutagenesis types of mutations with an accuracy of 93.88. The resulting system is useful for constructing a Gold Standard set of mutations extracted from the literature by human experts with minimal manual curation effort, providing direct pointers to relevant evidence sentences. Our system is able to recover mutations from the literature that are not present in state-of-the-art databases. Human expert manual validation of a subset of the literature extracted mutations conducted on 100 mutations from PubMed abstracts highlights that almost three quarters (72%) of the extracted mutations turned out to be correct, and more than half of these had not been previously annotated in databases.

## Background

Protein kinases are the most ubiquitous family of signaling molecules in human cells, accounting for approximately 2% of the proteins encoded by the human genome [1]. They can be further divided into sub-families that share significant similarity both at the sequence and structural level. A common feature of all kinases is their ability to transfer the terminal phosphate of ATP to serine, threonine or tyrosine residues of a target protein. Empirical studies also suggest a common catalytic mechanism whereby ATP and active site divalent cations are bound as well, and phospho-transfer is carried out by a shared set of amino acids. Despite these functional commonalities, experiments in yeast models [1,2] suggest that the protein kinase family as a whole is highly promiscuous, phosphorylating a range of different protein substrates, although individual sub-families may display a remarkable substrate specificity [3]. Kinases have a domain committed to the general function of catalysis, while another region (or even regions) are used in many cases to confer substrate specificity to the enzyme, without altering the general kinase folding, interfering with ATP binding or the general reaction mechanism. For reviews on the evolution of kinase structure and function see [4-7]. Several efforts have been made to provide a comprehensive access to information relevant to characterize human protein kinases through specific databases such as KinBase [1], KinMutBase [8] and MoKCa [9], or more general databases like PDB [10], PFAM [11] and CATH [12], storing information important to understand disease-association, functional and structural properties of kinases.

The relation of kinases with a number of diseases [13] and in particular with cancer [14-16] has prompted a number of large scale studies, in particular, Greenman *et al. *carried out the first large scale study of the variation associated with 518 protein kinase genes in 210 samples of cancer tissues and cell lines. Other HT studies not specifically restricted to kinases have obviously also contributed in understanding and providing information on mutations in protein kinases [14,15]. The interest of kinases and their implication in disease processes has continued with the study of Sjöblom and colleagues [15]. For a detailed review refer to Baudot *et al. *[17].

The sizeable amounts of information provided by large scale variation studies and the growing efforts of databases and resources to store and curate this information, are still not perfectly/completely connected with the many efforts dedicated to the detailed study of specific kinases in various biological systems [18] published in individual research papers. For instance, it is still a challenging task to establish for which individual mutations detected in HT studies there is already available information in the literature. Manual inspection and curation of specific variation studies and the exact linking to the textual information requires considerable resources.

Despite difficulties in extracting more complex language expressions referring to mutation mentions, regularities in describing mutations based on existing nomenclature conventions, promoted the implementation of automated information extraction and text mining systems for the identification of mutations in the literature [19-30]. Table 1 provides a short summary of previously published literature mining efforts for mutation information extraction.

**Table 1:** Literature mining approaches for mutation extraction. The additional materials sections (Additional file 1) provides a more detailed description of each method.

Collection of existing mutation extraction approaches		
**Method**	**Main characteristics and descriptive keywords of the approach**	**Ref**

MEMA	Uses regular expressions, gene and protein mention detection, co-mention proximity, OMIM validation	[19]
MuteXt	Uses regular expressions, GPCR and NR mention detection, co-mention proximity, sequence check	[27]
Yip *et al.*	Uses regular expressions, protein mention detection, SwissProt validation, extensive sequence check	[28]
CoagMDB	Uses regular expressions, serine protease mention detection, sequence check	[41]
Mutation GraB	Uses regular expressions, protein mention detection, graph shorted distance, sequence check	[20]
Mutation Miner	Uses regular expressions, protein mention detection, sentence co-mention	[21]
MuGeX	Uses regular expressions, protein mention detection, protein and DNA mutation disambiguation	[24]
VTag	Machine learning (CRF) detection of acquired sequence variations mentions (mutations, translocations, deletions)	[26]
OSIRISv1.2	Detection of human gene variations corresponding to SNPs	[42]
MutationFinder	Uses regular expressions and patterns, protein mutations, complex language expressions	[23]

Even though most of the existing manually curated mutation annotation resources are based on reading full text articles, existing automated systems mainly relied only on (subsets of) PubMed abstracts or a small collection of full text articles. To facilitate the interpretation of the biological implications and phenotypic effects of a given mutation, not only by clinical experts but also by database curators or for designing biochemical experiments (drug design and molecular functional studies) it is crucial to know whether a given mutation has been experimentally generated or is present in a naturally occurring sequence variation. This aspect has generally been neglected by previously developed approaches. Finally, only few systems were able to show results based on the combination of heterogeneous data derived from multiple information sources, derived from literature as well as based on experimental data generated by genotyping studies.

Here we examined the use of text mining methods to extract information from the literature about protein kinases and their specific mutations, link this information to the corresponding protein sequences from databases (normalization) and analyze how this information is distributed in protein kinases related databases and repositories. The results of comparing information from databases and text repositories are analyzed in terms of the quality of the information provided and the significance in terms of the knowledge related to PK structure and function. We therefore applied an available mutation extraction system, called MutationFinder [23] to the whole collection of abstracts contained in PubMed database as well as to a large set of automatically retrieved full text articles. To determine if a putative mutation mention really corresponds to a mutation or actually to something else we developed a module that allows filtering of false positive mutation mentions through a combination of named entity recognition, dictionary look-up and rule based methods. A supervised machine learning method relying on the SVM algorithm was used to score and classify based on its context whether a given mutation mention correspond to an experimentally generated (induced) mutation or is a natural sequence variant. A protein mention normalization system together with an mutation sequence checking approach was used to detect associations between mutations and human kinases co-cited in the literature. Validation and comparison to multiple existing mutation resources, including the SwissProt database [31] as well as the COSMIC [32], Greenman/Wood dataset of somatic mutations [14,16], KinMutBase [8], and SAAPdb databases [33]. The extraction and comparative mutation analysis were followed by a structural examination of the distribution of the different type of mutations in kinase regions of structural and functional importance.

## Results and discussion

**Figure 1:**
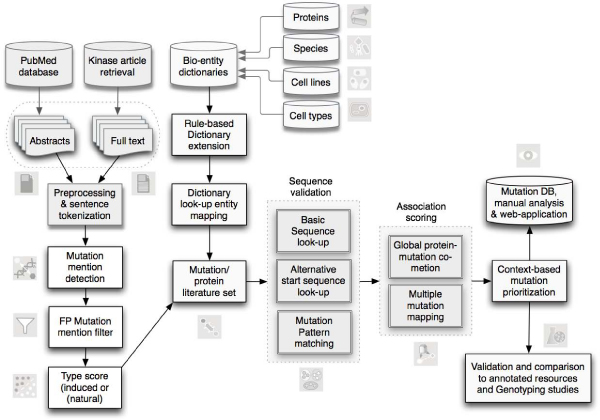
**Flow chart of the presented literature mining approach for mutation extraction**. This flow chart provides an overview of the different processing steps to extract mutations relevant for human kinases. The main steps include the construction of a kinase relevant article collection, the detection of mutation mentions, the scoring of the type of mutation (induced/natural variant), the linking of mutations to the corresponding protein sequence and the comparison to existing databases.

Here we present a workflow for extracting mutations within human protein kinases. The pipeline integrates article retrieval, detection of mutations mentioned in the literature, and a final validation of mutations linked to their corresponding protein sources. We carried out a comparative analysis of multiple annotation resources containing different mutation types. An overview of the resulting approach is presented in figure 1, illustrating the main steps of the mutation extraction pipeline.

### Systematic extraction and disambiguation of mutation mentions

For the initial extraction of single amino acid substitutions we applied the MutationFinder system, a modular software for point mutation recognition based on regular expressions and patterns detecting mutation mentions corresponding to residue abbreviations as well as other language expressions used to describe mutation events [34]. This system shows a competitive performance in terms of recall/precision when compared to other strategies [28] and has been evaluated using a manually generated Gold Standard collection of abstracts [23].

We applied the MutationFinder tool to the whole PubMed database (November 2008), resulting in the detection of 302,956 mutation mentions from 88,405 records, corresponding to a total of 61,329 unique mutation types (i.e. wild type residue, sequence position and mutant residue triplets). A more detailed analysis of the most frequent mutation types (see additional file 2), illustrated the importance of the Cystein to Tyrosine mutation in position 282 (C282Y, corresponding to the dbSNP:rs1800562) and the Histidine to Aspartic Acid mutation at position 63 (H63D, dbSNP:rs1799945), both occurring in the hereditary hemochromatosis protein (HFE, SwissProt:Q30201), known to be associated to several human diseases. These two mutations are mentioned over 3,500 and 1,900 times respectively. Some of the most frequent mutation types corresponded to cases of false positive (ambiguous) mutations mentions that actually consisted in names of cell lines (T47D cells) or mouse strains (G93A mouse model).

**Figure 2:**
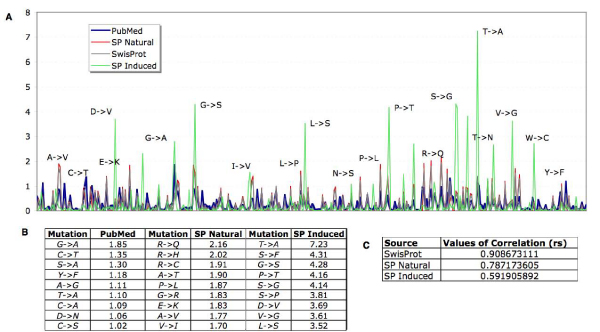
**Mutation type frequencies from PubMed and SwissProt**. A. Relative frequency of each mutation type derived from PubMed abstracts and from the SwissProt database. B. Most frequent mutation types from PubMed abstracts, and from SwissProt (SP), annotated as natural variant or induced (mutagenesis) substitutions. C. Values of the Spearman rank correlation between the text mining derived mutation types and the database derived mutation types. All p-values are below 10e-6, therefore statistically significant.

As the MutationFinder system was developed and evaluated using a collection of abstracts known to be relevant for mutations, primarily derived from citations related to mutant protein structures from the Protein Data Bank (PDB), we carried out a coarse level consistency analysis to determine how scalable this system is when applied to the whole PubMed database, where many articles do not necessarily resemble the data collection used for the initial system development. Assuming that the overall mutation types, contained in manually annotated resources like the SwissProt database should be similar to the ones encountered throughout PubMed we compared mutations extracted automatically from the literature to information contained in SwissProt, namely mutations being annotated as either natural variant, induced (mutagenesis) or both single amino acid substitutions. A comparative analysis of the frequencies of annotated mutation pairs (wild type residue and the associated mutation) showed that there are considerable differences between the mutations often encountered in naturally occurring variations as opposed to experimentally induced amino acid changes. The overall profiles resulting from the relative percentages of mutation types from different sources are similar (see figure 2A), despite existing differences encountered in terms of the most frequent mutations pairs extracted through text mining when compared to manual annotations (figure 2B).

**Figure 3:**
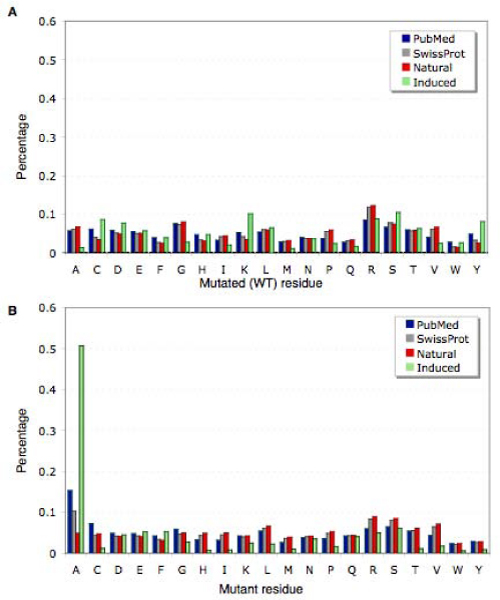
**Comparative analysis of wild type residues and mutations extracted from SwissProt and using text mining**. This chart illustrates differences in terms of wild type and mutant residue frequencies derived from the SwissProt database and obtained through automatic literature processing. A. Relative frequency of each wild type residue derived from mutations extracted from PubMed abstracts and from the SwissProt database. B. Relative frequency of each mutant residue derived from mutations extracted from PubMed abstracts and from the SwissProt database.

The most remarkable differences in case of the relative frequencies of mutation types extracted from PubMed compared to SwissProt corresponded to mutations formed by the residues, A, T, C and G (i.e. G-T, C-T, A-G and T-A substitutions). This is due to the intrinsic ambiguity of single letter mutation mentions that can correspond to both mutations at the DNA or protein level depending on the context. In order to distinguish between these two mutation levels, additional processing would be required. A more detailed analysis of the wild type residue and the mutant residue frequencies revealed that automatically extracted mutation residues are in line from what would be expected when examining the relative frequencies within a manually curated database (figure 3A and 3B).

The most frequently mutated residues mentioned in PubMed are Arginine, Glycine and Serine, corresponding also to the top ranking ones annotated in SwissProt. SwissProt shows more variability when comparing annotations of wild type residues from naturally to induced variations. In case of experimentally generated mutations, the residues most frequently replaced are Serines, Lysines, Arginines, Cysteines and Tyrosines, corresponding to functionally important residues. Considering the mutant residues, the literature mining extracted residues are consistent with the mutant residues from SwissProt, which shows great variation in case of Alanine induced and natural variant mutants. This can be explained by the widespread use of experimental approaches relying on the Alanine scanning method for identifying functionally relevant sites, as substitutions to Alanine usually still allow protein folding yet may give an altered phenotype. A more detailed description of the mutation disambiguation and filtering approach to remove false positive mutation mentions is provided in the method section.

### Scoring variation origin categories: artificial and natural mutations

**Figure 4:**
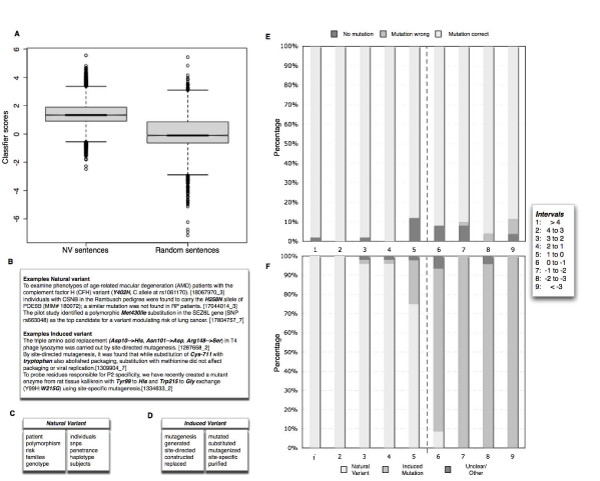
**Evaluation of classifying induced mutation mentions and natural variants**. A. Box plot of the sentence classifier scores for Natural Variant (NV) annotated mutations in SwissProt and a random subset of sentence scores from mutation mentioning sentences. B. Example cases of mutation mentions corresponding to natural variant and induced mutations. C. Example features used by the sentence classifier for the positive class (Natural Variant) and the Negative class (Figure D, induced mutation). E and F Manual classification result for 50 randomly selected mutation mentioning sentences for classifier score intervals. (1) Score above 4, (2) score range of 4-3, (3) score range 3-2, (4) score range 2-1, (5) score range 1-0, (6) score range 0 to minus 1, (7) score range from minus 1 to minus 2, (8) score range from minus 2 to minus 3, (9) score range below minus 3. Positive scores correspond to mutations classified as natural variant, negative scores correspond to mutations classified as induced/mutagenesis.

For extraction and management of biological annotations and to carry out functional analysis of mutations it is crucial to know the level of granularity and experimental context used for determining the phenotypic effect of a given amino acid substitution. The SwissProt database distinguishes here between induced or artificial (mutagenesis) and natural variant mutations, corresponding the former to less than 13 percent of the mutation annotations. Characterizations at the level of molecular functional implications and sub-cellular interactions of specific residues are commonly studied through experimentally induced amino acid changes. On the other hand associations to diseases such as certain cancer types and relevance for population groups or patients of a given mutation is usually studied by examining naturally occurring sequence variants. To address this important issue, allowing mutation mention scoring for each of these two basic categories of phenotypic descriptions we applied a supervised machine learning strategy for mutation sentence classification. We applied a SVM algorithm (using radial basis function as kernel) trained on a balanced sample set of 3,482 (71%) labeled sentences for induced and natural variant mutations and evaluated it on an independent test set of 1,400 sentences (29%), obtaining an accuracy of 94.64 (recall: 94.57 and precision of 94.71) on this collection. The size of the feature dictionary used by the classifier was of 11,803 word types (unique words, not stemmed). A manual inspection of the generated feature dictionary revealed that some of the relevant features corresponded to terms comprised in experimental techniques used to generate artificial mutations, such as site-directed mutagenesis. This basic evaluation schema is suitable to determine the performance on a controlled set of balanced instances, but does not take into account the actual distribution of the classes within a large collection of unlabelled data nor cases that even by human experts can not be clearly classified into one of these binary categories. Therefore we carried out both, a classification consistency analysis on the resulting sentence scores as well as a detailed evaluation and comparison against manually examined mutation sentences (see figures 4A to 4F).

To determine the overall classification and scoring consistency on the level of the whole mutation sentence collection extracted from PubMed described in the previous subsections, we analyzed the distribution of a database confirmed set of natural variant mutations against a randomly chosen set of mutation mentions. The first collection of sentences corresponded to mutation mentions cross-checked from SwissProt annotations as natural variants, resulting in a total of 10,886 sentences, none of these were contained in the original training nor test set. The second collection was constructed by randomly selecting an equal number of mutation mentioning sentences from PubMed abstracts. For each of these two sets we determined the corresponding sentence scores generated by the classifier. Figure 4A shows the box plot of the sentence classifier scores for the 10,886 natural variant mutation sentences and the equally sized random collection of mutation mentioning sentences. The scores of natural variant mutation sentences (mean of 1.37) were significantly higher when compared to the random subset (mean of 0.09), indicating that the overall scoring of natural variant mutation mentioning sentences derived from the independent SwissProt annotations are consistently higher than a random subset.

For practical purposes it is often useful to determine the actual performance of a system for a discrete set of score intervals or cut-offs, to enable a more efficient selection of instances for further examination or manual curation. Therefore we selected random subsets for sentence score intervals ranking from above 4 (positive class, natural variant relevant) to minus 4 (negative class, induced mutation or mutagenesis). Sentences of each of these sets underwent a two-step blindfold manual classification process to provide a more fine-grained analysis of the different aspects that might influence the actual systems performance. The first step consisted in classifying whether the sentence is mentioning a mutation or not to determine the effect of false positive mutation extraction. As a separate class we also recorded cases of mutation mentioning sentences where the directionality of the automatically extracted mutation event (wild type residue vs. mutant residue) was wrongly derived. When considering the mutation extraction performance across the score intervals for mutations classified as induced and natural variants, it seems that it was more difficult to correctly identify mutation mentions in abstracts that where close to the classification boundary or where scored as experimentally generated mutations.

The second step involved manually classifying mutation mentions into one of the following categories: (1) natural variant, (2) induced mutation or (3) unclear cases. We decided to add the latter category to take into account mutation mentions that even by humans could not be classified clearly into one of the two other types, either because the context of mention is not informative enough or because it is a truly ambiguous case. Figure 4E and 4F provide a detailed overview of the results obtained from this multi-step manual mutation classification for each of the score intervals. The classifier results are identical to human classification in over 95% of the cases for very high and very low mutation sentences score intervals, but drop to less than 77% for cases where mutations where classified as natural variants with classifier scores close to the classifier decision boundary (score interval of 1 to 0). Finally we also selected randomly a larger set of mutation mentions and carried out a bind fold manual labeling of these cases, to analyze the overall performance of the mutation classifier as well as to determine the distribution of natural variant and induced mutation mentions from PubMed abstracts. From the initially extracted set of mutations, 93.9% corresponded to correct mutation mentions without applying the mutation filter, compared to 97.0% when applying the false positive mutation detection step. Out of the correctly extracted mutations, 49.75% were manually classified as natural variant mutations, very close to the 47.2% of mutations classified as induced mutations. Surprisingly only 3.05% corresponded to unclear mutation types. Evaluating the sentence classifier results against the manually classified labels resulted in a precision of 93.88%, a recall of 91.09% (balanced f-score of 92.46) and an accuracy of 93.88, in line with the performance obtained with the previously used test set. An interesting false negative case was the sentence: The hexameric structure is important for protein stability, as demonstrated by studies with natural mutants (the Killer-of-prune mutant of Drosophila NDP kinase and the S120G mutant of the human NDP kinase A in neuroblastomas) and with mutants obtained by site-directed mutagenesis. In this particular case the authors refer to both natural variants as well as induced mutations, being S120G actually a natural mutation.

### Linking mutation mentions to human kinase sequences

Providing associations of mutations to their corresponding protein record and sequence is crucial to facilitate a more detailed characterization of structural effects of a given mutation and distribution within certain protein domains. This also allows direct comparison to functional annotations of proteins and mutations contained in manually curated annotation databases as well as to large-scale experimental results obtained by genotyping studies. Here we focus on associating the extracted mutations specifically to human protein kinases.

To obtain links between mutation mentions and human kinases we assumed that the corresponding protein names should be co-mentioned in the articles. After extracting all the mutation mentions from PubMed abstracts and a large collection of full text articles, these two data sets were processed for retrieving mentions of human protein kinases. In order to detect kinase protein mentions we applied a dictionary look-up approach, similar to strategies that participated successfully at the gene normalization task of BioCreative II [35]. To take into account inter- and intra-species protein name ambiguity, rather than using very strict protein-organism source co-mention criteria based on relative textual distances, we calculated for each article two scores reflecting (1) the contextual similarity of the article to the SwissProt protein record and (2) the overall association of the article to human species terms from the total set of tagged species terms.

This high recall protein normalization scoring strategy was followed by a more stringent sequence validation approach that allowed us to detect links of mutations and proteins by checking whether the actual mutation mention can be confirmed by looking them up at the protein sequence position. We restricted our analysis specifically to mutations occurring in the protein kinase domain, as defined in Kinbase [1]. A total of 567 triplets (i.e. article-mutation-protein associations) derived from abstracts could be validated by checking whether the extracted wild type residue was found at the mutated position in the protein sequence. In addition to this basic sequence look-up validation method we implemented five complementary mutation-sequence mapping strategies that take into account both, errors resulting from the wrong detection of the actual directionality of the extracted mutation with respect to wild type and mutant residues as well as inconsistencies and alternative sequence counting between the article and the database kinase sequence (see methods section). By applying this additional matching strategies we were able to recover 437 additional hits, corresponding to 43.53 percent of the total set of sequences validated protein mutation pairs. This resulted in a total of 1,004 triplets from 714 abstracts. In case of full text articles, the total number of triplets detected by the basic mapping was 3,911, being another 3,917 triplets recovered through additional sequence mapping methods. This resulted in 7,828 triplets from 3,496 full text articles. The average number of sequence validated mutations in the Protein Kinase Domain for abstracts was 1.41 and for full text articles 2.24, implying that often more than a single mutation is described in a given paper.

The global context of co-mention of kinase proteins and mutations defined by the multi-document collection where these co-occur, can be indicative for the actual importance of a particular mutation, being described and studied in various different paper. To use information provided by the corpus co-mention context, in addition to the total number of documents where the sequence validated mutation-kinase pair co-occurred we calculated the mutual information for each mutation pair.

### Comparison of text mining mutations to databases and genotyping studies

Several genomic studies (including a comprehensive analysis of all human kinases) have been dedicated to the characterization of mutations occurring in protein kinases in a variety of cancer tissues and cell lines. In these studies, a number of point mutations detected in somatic cell lines have been found to be associated with specific cancer types. The pathogenicity of these mutations depends on multiple factors related to the complex molecular environment in which protein kinase function takes place. Of special interest are mutations found within the protein kinase domain, as it is essential for the functional activity of these proteins. We therefore focused our analysis on automatic extraction of mutations mapped to this particular domain, common to all kinases, and carefully examined how they relate to previously characterized mutations retrieved from multiple databases and experimental high throughput genomic studies. We used the kinase domain definition followed by Kinbase [1], analyzing both the distribution of mutations within the Protein Kinase Domain, as well as the distribution of mutations according to the corresponding protein family topology. A total of 643 kinase domain sequence mutations were extracted from PubMed abstracts for a total of 128 different proteins. When considering the full text collection, we were able to increase considerable this number, obtaining a total of 6,970 mutation-protein pairs from 325 proteins. Using full text articles resulted therefore in a considerable increase of recovered mutations (more than 10 times more mutation-protein pairs when compared to abstracts) as well as being useful to increase the recall of proteins for which mutations had been extracted (more than doubling the initial number derived form abstracts alone). The increased recall for full text papers clearly justifies the computational effort required to retrieve and preprocess them.

**Figure 5:**
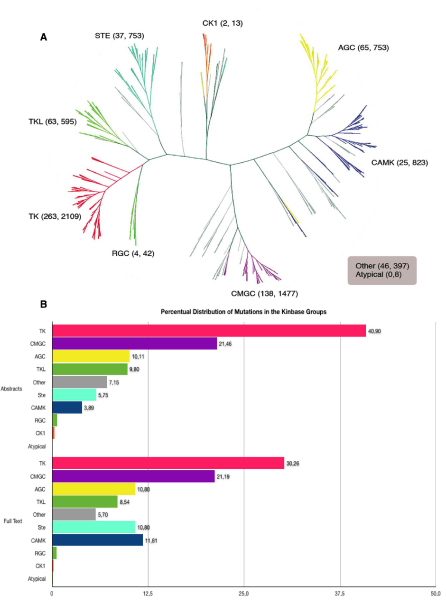
**Distribution of literature extracted mutations in the groups defined by Kinbase**. A. Number of mutations from the literature lodging in the different protein kinase domain groups in which Kinbase classifies the human kinome when the abstracts and the full text articles are taken into account respectively. B. Normalized distribution of mutations in the different protein kinase domain groups in which Kinbase classifies the human kinome when the abstracts and the full text articles are taken into account respectively

Figure 5A shows the distribution of the mutations extracted from the literature into the different groups in which Kinbase [1] classifies the protein kinase domain of the human kinases. For a more detailed description of the different kinase groups refer to the methods section. Although there are differences in the number of mutations present – with more than a half of the mutations either within the TK or the CMGC clades – all the main groups are represented in the results both when PubMed abstracts or full text articles are taken into consideration. Figure 5B depicts the normalized distribution of mutations in the different protein kinase domain groups in which Kinbase classifies the human kinome when the abstracts and the full text articles are taken into account respectively. It is evident that no matter which dataset is used, either abstracts or full-text articles, the distributions are very similar independently of the very uneven absolute numbers between both datasets, which confirms that complementary results are provided by the two very different approaches.

#### Confirmation and comparison to experimental and curated data

In order to assess whether the mutations recovered from the existing literature by our system were already present in commonly used databases or are newly recovered instances, we herein studied the overlap between the mutations in the protein kinase domain both in the databases and the results from our extraction pipeline. Table 2 represents the percentage of each database covered by the Text Mining results.

**Table 2:** Overlap between the different knowledgebases and the literature extracted mutations

Literature derived mutations and overlap with knowledgebases				
**Knowledgebase (KB)**	**Total Mutations in KB **[weight]	**Abstract**	**Full Text**	**Combined (Abs+FT)**

**SwissProt – all**	710 [56.13%]	134 (18.87%)	328 (46.20%)	365 (51.41%)
**SwissProt – natural variant**	459 [36.28%]	99 (21.57%)	196 (42.70%)	230 (50.11%)
**SwissProt – mutagenesis**	251 [19.84%]	35 (13.94%)	132 (52.59%)	135 (53.78%)
**SAAPdb – all**	610 [48.22%]	65 (10.66%)	106 (17.38%)	125 (20.49%)
**SAAPdb – pathogenic deviations**	323 [25.53%]	64 (19.81%)	105 (32.51%)	123 (38.08%)
**SAAPdb – neutral**	287 [22.69%]	1 (0.35%)	1 (0.35%)	2 (0.70%)
**Greenman & Wood**	254 [20.08%]	4 (1.57%)	12 (4.72%)	13 (5.12%)
**Greenman & Wood – driver**	119 [9.04%]	3 (2.52%)	9 (7.56%)	9 (7.56%)
**Greenman & Wood – passenger**	135 [10.67%]	1 (0.74%)	3 (2.22%)	4 (2.96%)
**COSMIC**	200 [15.81%]	4 (2.00%)	11 (5.50%)	12 (6.00%)
**KinMutBase**	83 [6.56%]	32 (38.55%)	32 (38.55%)	43 (51.81%)
**All Databases**	1265	148 (11.70%)	354 (27.98%)	399 (31.54%)

#### Recovery of disease associated mutations: overlap with KinMutBase

KinMutBase [8] is a manually curated knowledge base for human disease-related mutations in protein kinase domains. At the time of the study, March 2009, a total of 83 single-point pathogenic mutations in the protein kinase domain of 10 different proteins were provided in KinMutBase. We were able to confirm 32 (38.55%) of these mutations from abstracts and also the same number from full text articles. When combining the mutations from both article collections we recover more than half of the mutations from KinMutBase, namely a total of 43 mutations (51.81%). This suggests that the mutation mentions from both document collections are essentially complementary, and some of them could only be detected in one of the document sets.

#### Recovery of natural variant and induced mutations: overlap with SwissProt database

The Swissprot Variant database [31] provides experimentally-verified information about mutations present in UniProtKB, containing a set of 710 mutations in the protein kinase domain of 194 different proteins. 251 (35.35%) of them corresponding to mutagenesis experiments whereas 459 (64.65%) correspond to reported natural sequence variants. Using our text mining approach we were able to recover 134 (18.87%), 328 (46.20%) and 365 (51.41%) of the mutation contained in the database when the abstracts, the full texts and the combination of both was used, respectively.

When considering the overlap of extracted mutations with respect to each of these two mutation type classes (natural variants and mutagenesis) we were able to obtain similar percentages for both groups from the combined article collection, 50.11% of the mutations annotated as natural variant and 53.78% of the mutations annotated in SwissProt as mutagenesis.

Interestingly, we found differences in the overlap percentages of recovered mutations from abstracts and from full text articles when looking at these mutation classes individually. When considering the mutations derived from abstracts, 21.57% of the natural variant annotated mutations could be detected, as opposed to only 13.94% of the mutagenesis annotated mutations. The opposite trend was observed in case of full text articles, where we extracted 52.59% of the induced mutations and 42.70% of the natural variant mutations. This suggests to certain extent that experimentally induced mutations annotated in SwissProt are usually not mentioned in abstracts, but rather in full text articles.

#### Recovery of structurally important mutations: overlap with the SAAPdb repository

The SAAPdb [33] is a resource for the analysis and visualization of the structural effects of mutations. At the time of this study, SAAPdb contained 610 point mutations located in the protein kinase domain of 230 proteins. 52.95% (323) of the information corresponds to mutations previously reported as pathogenic deviations (PDs) whereas the rest corresponds to neutral SNPs (287, 47.05%).

Our system recovered 65 (10.66%) and 106 (17.38%) of the mutations previously stored in the database when the abstracts and full text articles were taken into consideration. For the joint abstracts-fulltext dataset, 125 (20.49%) of the mutations present in SAAPdb were found.

With regard to the pathogenicity of the mutations found, for the particular case of the combined dataset, we were able to find 123 (38.08%) of the pathogenic deviations, whereas only 2 neutral SNPs were recovered. This highlights the fact that the literature is biased towards those mutations known to be functionally active and harmful for the individual. It is interesting to remark that none of the other databases analyzed contained records for the neutral SNPs in SAAPdb either.

#### Recovery of somatic mutations: overlap with the Greenman and Wood dataset

The Greenman and Wood dataset was built from the results shown in the original papers [14,16] by the authors where they report 254 somatic mutations corresponding to the protein kinase domain of 164 proteins in diverse human cancers. In addition, the mutations are sub-classified according to the pathogenic character predicted into drivers (cancer associated somatic mutations) and passengers (neutral mutations). The contribution of each class to the whole database is 46.85% and 53.15% respectively.

Our system recovers only a very small fraction of these somatic mutations since only 13 (5.12%) of the instances in the dataset were able to be recovered in the best case scenario, where the combined article set (abstracts+fulltext) was used. This means that only a small proportion of the mutation dataset detected by experimental High Throughput approaches could be linked directly to other literature evidences. Our system recovered 9 (7.56%) driver mutations versus 4 (2.96%) passenger mutations. A very similar trend was observed for the case of the COSMIC database, which shares around 95% of the information contained in the Greenman/Wood dataset for the particular case of the protein kinase domain.

#### Result summary and structural mutation distribution

Finally, we wanted to assess how many of the mutations we were able to recover from the total set of mutations in the 5 studied datasets (namely, SwissProt database [31] as well as the COSMIC [32], Greenman/Wood dataset of somatic mutations [14,16], KinMutBase [8], and SAAPdb databases [33]) in order to get a view of the coverage of the existing knowledge by our method. To do so we built a non-redundant set with 1265 mutations in 317 different kinases. The different databases are unevenly represented, and the weight of each database is reported in the last row of Table 2, where the overlap between the different databases is assessed, under the epigraph 'All Databases'.

Out of the 1265 mutations in the combined database, 148 (11.70%) were found by the Text Mining approach when the Pubmed abstracts were scanned. By contrast 354 (27.98%) mutations were recovered when the full-text articles were taken into consideration, and 399 (31.54%) when the combined abstracts+fulltext dataset was used. The increased recall of this combined method clearly justifies the computational effort required.

**Figure 6:**
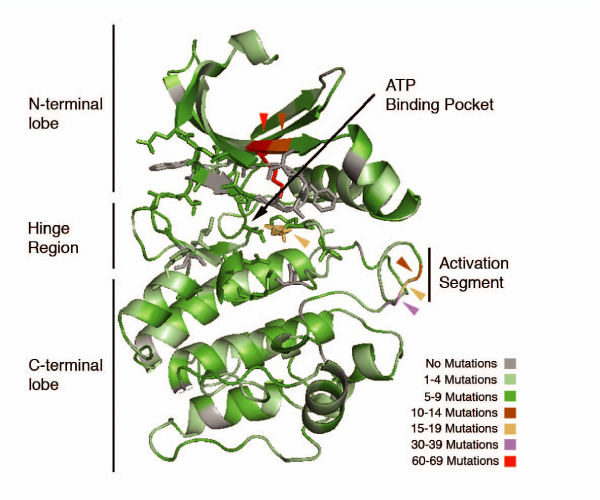
**Localization of the mutations extracted from the Pubmed abstracts within the structure of the Protein Kinase domain**. The ATP binding pocket is represented with sticks. The DFG motif (activation segment, essential for kinase function) allocates a big number of mutations. The light brown Asparagine (central part of the figure) in the inter-lobe region, more than 10 mutations. The highest density residue is Lysine 64 (red), allocating 65 mutations. This residue has been reported as essential for protein function and ATP binding. We observe that most of the mutations allocate in or near the ATP binding pocket or the activation segment and that mutations outside the binding pocket correspond generally to low mutation density residues (colored in grey and green in the kinase domain model).

Although there are mutations scattered everywhere in the kinase domain structure, a considerable mutation density is encountered close to functionally relevant parts of the protein, i.e. the ATP binding pocket, the DFG motif in the activation loop. Figure 6 shows a detailed view of the mutation density distribution within the protein kinase domain model. ATP binding Lysine 64 shows the highest density of mutations, with a total of 65 mutations, followed by residues forming the activation segment (up to 39 mutation per residue) and several residues conforming the ATP binding pocket.

### Worked example: the Epidermal Growth Factor Receptor

The interest of the system presented here is not only that the user can gather mutations from the literature that are not reported in the databases, but also that one can get a summary of sentences mentioning those mutations that will help to assess the pathogenicity (and in the best possible scenario, the function) of the mutations newly discovered. A working example is provided here: Mutations in the EGFR.

The epidermal growth factor receptor, also known as EGFR, is a protein kinase involved in the control of cell growth and differentiation which has been reported of interest in the development of breast cancer since binding of EGF to its receptor leads to dimerization, internalization of the binary complex, induction of the tyrosine kinase activity, stimulation of cell DNA synthesis, and cell proliferation.

There are several well-known mutations reported for this protein in current state-of-the-art databases storing information on mutations (SwissProt [31], COSMIC [32], Greenman/Wood [14,16], KinMutBase [8], SAAPdb [33]) Even more, for some of them, their involvement in disease has been investigated and annotated in the corresponding databases. For instance, the somatic mutations G719S, L858R and T790M have been previously reported in relationship with lung cancer [16,36].

By contrast, our system was able to recall from the literature 32 mutation mentions that have not been reported in the dedicated databases. In order to better understand the effect of these mutations, our approach is also capable to provide context information that can be used for the interpretation of the role played by the mutations as described herein.

To provide an example, in the case of Y845F (transformed to Y869F due to the presence of a signal peptide) we were able to find the following sentences 'Furthermore, transient expression of a Y845F variant EGFR in murine fibroblasts resulted in an ablation of EGF-induced DNA synthesis to nonstimulated levels.' (PMID:10075741), 'Stably transfected B82L cells with a point mutation of the EGFR at Tyr-845 (B82L-Y845F) exhibited only basal Ras activity following exposure to Zn2+' (PMID:11983694), 'In contrast, LPA-elicited DNA synthesis and migration were augmented in cells expressing EGFR, EGFR(K721A), or EGFR(Y845F), but not EGFR(Y5F), although the PDGF responses were indistinguishable' (PUBMED 15364923). The information retrieved suggests the involvement of Tyrosine-845 from EGFR in DNA synthesis via binding to EGF.

In addition, the system also retrieves functionally neutral results that are often discarded and not stored in the databases although they contain very useful information for the contextual interpretation of the involvement of point residues in protein function 'Unexpectedly, the Y845F mutant EGFR was found to retain its full kinase activity and its ability to activate the adapter protein SHC and extracellular signal-regulated kinase ERK2 in response to EGF, demonstrating that the mitogenic pathway involving phosphorylation of Y845 is independent of ERK2-activation' (PUBMED 9990038). The structural model of this protein together with a summary of the residue and mutation information is included in additional material file 3

## Conclusion

In this paper we presented the first approach to extract human kinase mutations from both PubMed abstracts and a large collection of full text articles, comparing the obtained results to mutations that have been manually curated from the literature by annotation databases as well as data generated by genotyping studies. Automated mutation extraction can assist manual curation efforts by providing direct pointers to mutation evidence sentences for quick manual examination. The MutationFinder system was useful to detect mutation mentions from both abstracts and full text articles combined with some additional filtering of ambiguous mutation mentions. Some potential future improvements of this basic mutation extraction system could consider wrongly extracted mutation mentions resulting from mentions of sequence ranges or the inclusion of detection of stop codons (e.g. R97X). Several strategies have been used to filter ambiguous mutation mentions and to discriminate between mutations at the level of DNA and proteins. We carried out a detailed consistency analysis of the mutations detected by means of literature mining to the content of manually curated annotations. Future steps could include a more detailed exploration of the actual reliability scoring and ranking of sequence validated mutations through the use of: (1) mutation-protein proximity analysis in full text articles, (2) species and organism source ambiguity examination and (3) analysis of the probability of finding a given mutation within the target sequence per chance, considering the actual residue composition of proteins and kinases. By using a standard machine learning approach we were able to score the level of phenotypic description based on contextual information provided for a given mutation, classifying each mutation mention as induced (artificial, generated by mutagenesis experiments) or natural variant (polymorphisms, SNPs and somatic mutations). This aspect is especially important as it connects mutation relevant information generated by different scientific domains, i.e. data generated by clinical, epidemiological and human genetics studies with molecular biology and biochemical in vitro experiments. Extraction of mutation information from multi-document collections is useful to complement different scientific discoveries and characterizations described across various papers, increasing thus efficiency in relating entries to each other and integrating multiple complementary evidences discovered by different research groups. Problems related to sequence shifts or cases of so-called sequence conflicts when comparing the numbering used by article authors to the sequences contained databases like SwissProt were addressed by using various sequence validation strategies, from the basic residue look-up to the use of text derived sequence patterns. These Sequence conflicts can be the result of sequencing errors, sequence variants or isoforms that are not well characterized or even from alternative counting when considering N-terminal signal peptides [28]. To resolve such sequence conflicts is even a cumbersome task for human experts. We can recover 7,184 potential mutations on kinases in the Protein Kinase Domain from text (643 from abstracts and 6970 from full text). Information from abstracts and full text is essentially complementary, as sometimes the full text article for a mutation mentioning abstract is not available or even written in another language different from English. Although some of the extracted mutation-kinase associations might be erroneous, they still provide a very good basis for additional annotation efforts, in some cases valuable for the in depth analysis of specific proteins (as in the example shown here).

**Figure 7:**
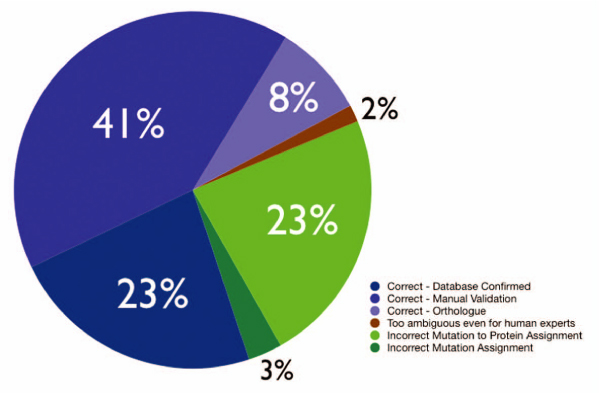
**Success estimate of the extraction pipeline by human expert manual validation**. These percentages were calculated upon a manual sampling and validation protocol conducted on 100 abstracts. Correct – Database confirmed: These are the mutations that have been found already in at least one of the analyzed databases (Uniprot, SAAPdb, COSMIC, KinMutBase or Greenman). Correct-Manual validation: This subset corresponds to the mutation-protein pairs that have been found correct after manual validation on 100 abstracts. Correct – Orthologue: This subset corresponds to the cases where mapping is confirmed by manual validation and the mutation is mapped to a non-human orthologue. Incorrect Mutation to Protein Assignment: Corresponds to the cases where both proteins share the same amino acid at the mutated position and the algorithm choses the incorrect pair. Incorrect Mutation assignment: Cases where the mutation is not properly identified. An interesting particular case are the confusion with cell lines (accounting 66% of this category) Too ambiguous even for human experts: Odd little informative cases where even human experts reading the abstracts are not able to identify to which protein the mutation corresponds to.

Interestingly only a very minor fraction of the mutations detected in hight throughput genotyping studies [14,16] correspond to previously identified mutations mentioned in the literature. As a considerable number of these HT generated data correspond to mutations that do not have any deleterious effect, it is understandable that they lack further careful characterization published in the literature. In general we find that 31.54% of the mutations contained in manually annotated databases can be directly recovered from papers, important for assuring the database-literature coherence. The remaining mutation records lack direct evidence about its origin in text, potentially due to (1) missing accessibility of the corresponding full text articles or additional materials (especially in case of older publications), (2) general limitations in terms of recall of mutation mention extraction methods or (3) limitations in the protein normalization and mutation to sequence associations. We estimate that, based on the proportion of natural and artificial variations described in the literature, a considerable fraction of the text mining derived mutations not contained in any of the existing kinase mutation resources might correspond to experimentally generated induced mutations. From a manual inspection of natural variant mutations we were able to differentiate between four main mutation types, some of them not considered as annotation relevant by existing databases but nevertheless important for interpreting the practical relevance of individual mutations, these include: (1) mutations with no clear association to the studied disease phenotypes, (2) mutations that are protective against some pathological condition, (3) mutations that are deleterious and that promote the pathological condition (e.g. increased disease risk). On the symmetric view 5.55% of the automatic literature annotations (23.02% from abstracts and 5.08% from full text) correspond to database confirmed entries, implying that a considerable fraction of the extracted mutations through literature mining is potential new information still to be annotated. In order to assess to which extent this new information can be trusted a human expert manual validation protocol was conducted on a randomly selected sample of 100 mutations taken from mutation mentioning abstracts (see Figure 7). We demonstrate in this work how the power of text mining combined with bioinformatics approaches can be used to discover and link information in key areas of biology, being able to result in a framework for supporting manual mutation literature curation and with the potential to adapt an analogous pipeline to other protein families going beyond the kinase/mutation analysis. Our work resulted in a collection of kinase mutation literature links (mutations, positions, sentences) derived form both full text articles and abstracts. Our work shows that extraction of mutations from full text articles is feasible and that it could be applied to the whole set of full text articles from PubMed records in case these access to those is provide din the future.

The experiment shows that for 23% of the mutations there was a positive confirmed record in at least one of the analyzed knowledgebases (SwissProt [31], COSMIC [32], Greenman/Wood [14,16], KinMutBase [8], SAAPdb [33]), being consistent with the results previously shown for the automatic extraction pipeline. In addition, and an important added value provided by our system, 41% of the results were correct assignments between the protein and the mutation extracted by text-mining that were not reported in the knowledgebases. Finally, 8% of the mutations corresponded to orthologs having the same amino acid that the human protein at the specified position, which can be considered positive hits as well, as they essentially represent information generated for human kinases using animal models. In summary, we estimate that almost three quarters (72%) of the extracted mutations correspond to positive hits being either previously annotated mutations, correct novel mutations or mutations of close orthologs.

Interestingly, a small proportion of the records (2%) were too ambiguous even for human experts, lacking enough information even to perform manual validation.

## Materials and methods

### Sequences of protein kinase domains using KinBase

The KinBase resource (, [1]) is a repository storing the currently accepted classification of eukaryotic protein kinases, which are categorized into two main groups: 'conventional' protein kinases (ePKs) and 'atypical' protein kinases (aPKs). The ePKs form the largest group and they have been subdivided into eight groups by sequence similarity of the catalytic domains, the presence of accessory domains, and by considering different modes of regulation. The eight ePK groups defined in KinBase are: the AGC group (including cyclic-nucleotide and calcium-phospholipid-dependent kinases, ribosomal S6-phosphorylating kinases, G protein-coupled kinases and close relatives of these kinases), the CAMKs (calmodulin-regulated kinases); the CK1 group (casein kinase 1 and close relatives); the CMGC group (including cyclin-dependent kinases, mitogen-activated protein kinases, CDK-like kinases and glycogen synthase kinase); the RGC group (receptor gyanylate cyclase kinases); the STE group (MAP Kinase cascade kinases), Tyrosine kinase group (TKs); and the TKL group (Tyrosine kinase like family) which are a cluster of serine-threonine kinases resembling TKs. Another broad, miscellaneous group called 'other' is also considered for those proteins that do not fit in any of the predefined sets.

At the time of the analysis, KinBase contained 620 human protein sequences of which 518 correspond to protein kinases not considered to be pseudogenes. Although kinases described as pseudogenes are transcribed and might even have a residual or scaffolding function, kinase pseudogenes were not mapped onto Uniprot (SwissProt/Trembl) since many of them are partial transcripts or have stop codons in their sequence. Since KinBase does not directly map its entries onto Uniprot, this mapping was performed using a BlastP [37] search for each kinbase sequence against a custom database containing all entries in Uniprot annotated as human protein kinase domain. Once the mapping was performed, we were able to map 488 Kinbase identifiers to a valid Uniprot entry, 474 of them (97.13%) at sequence identity levels of at least 95%.

### Mutation extraction from abstracts and full text articles

The used mutation extraction pipeline has been applied to two text data sets, one consisting in the whole collection of PubMed abstracts, and the other in a set of 19,404 full text articles. The full text articles were automatically downloaded using an in house full text retrieval system that had previously been implemented. To prioritize full text articles for download, three different criteria were considered. The first selection criteria was based on information contained in the corresponding abstracts, such as mention of mutations, mention of human kinase proteins and a combination of keywords (including 'human kinase mutation'). The second selection criteria was based on extracting all the PubMed references for human kinases contained in multiple databases (e.g. SwissProt, MINT, IntAct). The third selection criteria was based on analyzing the fraction of mutation mentioning abstracts for each journal, prioritizing a set of journals (and thus their articles) for retrieving their full text articles. These journals included: the American Journal of Human Genetics, European Journal of Human Genetics, Human Genetics, Human Mutation and Human Molecular Genetics. Each of the full text articles was automatically converted into plain text using pdftotext. Both abstracts and full text articles were then preprocessed applying an in house rule-based sentence boundary detection system that we optimized for PubMed abstracts. We applied the MutationFinder system two both the full text and abstract sentence collections using a cluster of 64 Mac PPC G5 processors running Darwin.

### Mutation disambiguation and filtering

The performance of information extraction methods that detect mutation mentions from the literature is affected by the underlying article selection criteria used. When applied to the whole PubMed database, a fraction of extracted mutation mentions are ambiguous, and therefore can, depending on the context correspond to a range of other bio-entities, like cell lines, protein names or clones. Only few previously published approaches did a more careful examination of wrongly extracted mutation mentions, most of these ambiguous mentions correspond to single letter mutations. Horn *et al. *compiled manually a list of exceptions to avoid mislabeling of other phrases as mutations, examining also certain terms co-mentioned in the context (e.g. cell line, tumour or cancer). For filtering single letter mentions that might correspond to mutations at the level of DNA or RNA they analyzed words surrounding the point mutation, but did not provide further details regarding this process [27]. Erdogmus and colleagues addressed DNA versus protein mutation disambiguation through a supervised learning approach based on the Naïve Bayes algorithm, they prepared a collection of 2,771 mutation mentions at the protein level and 768 at the DNA level and obtaining an accuracy of 84.7 [24].

We propose an approach for targeted mutation pattern sense disambiguation and filtering of mentions that do not correspond to protein mutations. Therefore we examined manually a large collection of mutation mentions to determine the sense inventory with respect to the context of occurrence, discriminating the main classes of false positive ambiguous mutation mentions and characterizing their semantic categories. The majority of these corresponded to one of the following three semantic types:

• Cell lines or cell types. There are several frequently mentioned cell lines that resemble mutation mentions. Among these are the human glioblastoma cell line T98G, the T-cell line M14T, the adrenocortical cell line H295R or other commonly used cell lines such as T47D or T24C.

• Taxonomic entities. Certain taxonomic names, especially bacterial strains, cloning vectors and certain animal models (e.g. mouse strains) contain words that are similar to single letter mutations. Example cases include the strains: E. coli K12S, A. viscosus T14V, P. pneumoniae R36A, A. naeslundii T14V, Mycoplasma sp. G145T or the yeast strain S288C. Also clone identifiers (e.g. W12I and W12E) or plasmids (e.g. E. coli plasmids P15A) can result in false positive mutation hits. A special case of ambiguous mutation mentions is encountered in transgenic mouse models like G93A transgenic mice. It consists in a mouse strain expressing a G93A mutant form of human SOD1 protein, but usually is mentioned as the name of the strain rather than as a reference to this particular mutation.

• Protein and gene names. Several protein names do match the patterns used to identify mutations from the literature, although some of these correspond to human proteins like S100D and S100E, a considerable fraction are viral gene names (e.g. A10L of the vaccinia virus, A11L variola virus or the poxvirus protein A52R).

We found some additional cases of wrongly tagged mutations that could be classified as drugs or compounds (e.g. the antibiotic A83586C, the immunogen A27L or the antifungal antibiotics A9145C). To determine the semantic class of a given mutation occurrence we explored the use of knowledge-based methods relying on machine-readable dictionaries (MRDs) for sense disambiguation based on local context analysis. In order to address this disambiguation task we assumed (1) One sense per discourse, namely that within a given document the target mutation mention is consistently used as either a mutation or one of the three other semantic types previously introduced; and (2) One sense per collocation, implying that nearby co-mentioned words provide strong clues to the sense of the target mutation mention.

Three lexical resources were compiled for taxonomic entities, protein/gene names as well as cell lines. Due to limited lexical coverage of cell line information in existing biological ontologies such as the Cell Type ontology, we generated automatically a cell line dictionary through use of a named entity recognition method (ABNER, [28]) applied to mutation mentioning PubMed abstracts. This resulted in a total of 9,252 cell line names, out of which 1,124 corresponded to mentions that could potentially match mutation patterns. We incorporated from the list of cancer cell lines contained in the COSMIC database five additional names resembling mutations. This cell line dictionary was used to filter ambiguous mutation mentions (over 13,500 sentences). We also generated automatically 922 pattern templates based on multi-word cell line names, where the original word resembling a mutations is used as a slot to be filled with ambiguous mutation mentions (see table 3).

**Table 3:** Mutation disambiguation patterns.

Example cases of Mutation disambiguation dictionary records and patterns			
**Cell lines patterns**	**Cell lines names**	**Proteins/Genes names**	**Taxonomy names**

human glioblastoma cell line MUTATION	breast cancer T47D cells	Met-1 serine protease	Aeromonas sp. F713E
MUTATION glioblastoma cell line	T98G human malignant glioma cells	S100C	Bacillus sp. G100I
MUTATION control cells	L5178Y lymphoblasts	Sperm surface protein P34H	Candida sp. N12C
MUTATION cultured cortical neurons	human cervical cancer C33A cells	R18I.1	Synechococcus sp. D120S
T3 MUTATION preadipocyte clones	-BRAF (V600E) thyroid cancer cells	Protein C184L	Symbiodinium sp. H10K

For taxonomic entities we assembled a dictionary of species names derived from the NCBI Taxonomy and used a dictionary look-up approach with these names for filtering potentially ambiguous mutations. A total of 584 taxonomic names (and their variations) contained words matching mutation regular expressions, most of them from cloning vectors and bacterial strains. Out of these we generated 128 disambiguation patterns for taxonomic mentions. A similar approach was followed for disambiguation of mutations matching protein and gene names, relying on a protein dictionary extracted from the UniProt database. The total number of protein and gene names from UniProt matching mutation mentions was 295. These were exploited for generating 29 disambiguation patterns that were manually revised to remove too general patterns, resulting finally in a set of 25 patterns.

A special case of ambiguity is encountered when distinguishing between mutations at the level of DNA, RNA and protein sequences. To enable discrimination between these different mutation types official nomenclature guidelines state that the description should be preceded by a letter indicating the type of reference sequence, p in case of protein sequences (e.g. pCys76Ala or p.C76A), g for genomic sequences, c for cDNA, m for mitochondrial sequences and r for RNA sequences [38]. Unfortunately in practice these standards are not sufficiently followed resulting commonly in ambiguity at the level of the corresponding reference sequence type, which requires a specific disambiguation strategy. This scenario is somehow similar to the distinction between gene and protein mentions, where even for human experts it is sometimes challenging to make clear decisions.

To handle the automatic distinction between DNA and protein mutations, we explored the use of different selection criteria that humans actually follow to achieve this task. We applied a hand crafted rule-based technique, with the implicit advantage that it does not require the construction of large training collections of representative sample cases for different types of DNA/protein ambiguous mutations. As contextual representation for disambiguation of mutation patterns we used: (a) implicit information from the mutation itself, i.e. mutation sequence position, (b) features derived from the local context, i.e. words enclosed in the corresponding sentence, and (c) distant content words from the whole abstract as contextual cues, i.e. other co-occurring mutations.

A useful characteristic to distinguish mutations at the DNA and protein level is actually provided by the mutation position number. The average length of sequences in UniProt is 360 amino acids, being the longest sequence 35,213 (the Titin protein from mouse). When looking at the mutation positions annotated in SwisProt, 96.76% are below 2000, 98.72% are below 3000 and 99.25% are below 4000. Therefore a basic aspect that we explored here was to filter mutations by position numbers allowing three positional cut-offs (2000, 3000 and 4000). Example cases of DNA mutations that can be successfully detected with this simple criterion are T1191C (PMID 15993850), G2950692A (PMID 15862761) and G20210A (PMID 18501222).

The local context of a given mutation mention, represented by the sentence in which it occurs can provide hints towards the mutation type. We generated two lists of terms that are associated either to mutations at the level of proteins or DNA based on manual inspection and extension of the features used by a sentence classifier trained on a small sample set of 687 DNA and protein mutation mentions. We used terms from these two lists mentioned within the mutation sentences to calculated the overlap coefficient of Lesk for scoring them as DNA or protein associated [39].

Certain distant content words co-occurring with a mutation in the whole abstract can be used as contextual cues for disambiguation. Here we explored the use of other co-mentioned mutations to determine the cooperative effect for mutation disambiguation, under the assumption that if multiple mutation patterns co-occur, and all of them resemble DNA mutations, it is consequently more probable for each of them to corresponds to a DNA rather than a protein level mutation. From manual examination of the resulting hits, we determined that at least 4 distinct mutations had to be co-mentioned in a given abstract, and that at least two different mutation combinations were needed (to avoid filtering of systematic Cys to Ala-scanning mutations). An example case illustrating this idea is the PubMed record 9240741, where all the following mutations are co-occurring: T1448C, T1366G, G1604A, A1226G. Finally we also took into account the numerical relation underlying the codon triplets and their encoding for amino acids as filtering criterion for cases where for a given ambiguous mutation, another co-mentioned mutation fulfill the positional information condition: position of DNA ambiguous mutation is equal to 3 times the position of a co mentioned mutation, as illustrated for C684G and N228K in: The novel mutations include T302C (L101P), C684G (N228K), and G1063C (A354P) (PMID 9889017).

### Mutation phenotype level classification: natural and induced

The classification of mutation mentions into natural variant or induced mutations was carried out using a sentence classifier approach using words co-mentioned with the mutation within the sentence. We used a SVM implementation (SVMLight, [40]) with radial basis kernel function (default parameters) which explored several feature weightings, finally using term frequency in order to avoid inconsistencies resulting from the class balance when weighting the used features. The initial feature dictionary was filtered using an in house stop word list (See additional file 4). We carried out also additional word filtering to remove numerical expressions and words with a length below 3 characters. The training set of sentences was derived from mutations of proteins extracted from papers and then cross checked using the SwissProt database whether they corresponded to natural variant or mutagenesis annotations.

### Protein and species mention detection

For the detection of protein and organism names we used a dictionary look-up and maximum sub-string matching algorithm implemented in C and Perl. The initial gene and protein dictionary of human kinases was extracted from SwissProt and automatically extended using heuristics and rules taking into account common typographical variations encountered in gene and protein names and symbols. These covered aspects related to the use of hyphens (generating variants with hyphens, with white space and without white space), capitalization (generating variants in upper case letters and capitalized versions) and word ordering. This resulted in a human kinase protein dictionary of 2,582,220 protein name-database identifier associations. This dictionary was further manually processed based on the information content of each tagged protein mention to remove some highly ambiguous protein name variations.

### Mutation sequence validation

To associate co-mentioned proteins and mutations from a given article, previous efforts [19,27] often considered local text associations in terms of distances between a mutation and the nearest mentioned protein (proximity scores). These document-centric associations have clear limitations in terms of the performance, and therefore recent efforts tried to improve the underlying performance through looking up the mutation at its corresponding position within the protein sequence. In an effort to increase the recall of the method we implemented a cascade of several strategies for mutation sequence validation that included the following strategies: (1) Sliding window algorithm that searches for a pattern of mutations along the sequence instead of exact position – using the numbering given in the mutation – co-occurrences in the sequence. The algorithm iteratively scans each position in the sequence and searches for co-occurrences of the other mutations mentioned in the same abstract in positions relative to the starting one giving priority to the distance, in terms of sequence, between al the mutations in the same abstract instead of the exact positions provided. The main capability of this approach is that is able to deal with the different means in which the starting position of a protein can be defined, the most graphic case being the presence – or not – of a signal peptide but other examples can be provided (sequencing errors or discrepancies, inclusion of promoter regions, and so on. Since the finding the profile by chance is quite easy for trivial results (the easiest of them all being patterns consisting of just one mutation) a limitation in the complexity of the pattern was established, being taken into consideration only those patterns having at least 3 mutations at different sequence positions. (2) Basic mutation to sequence position mapping: looking up the wild type residue of an extracted mutation mention in the corresponding protein sequence position. (3) Alternative mutation directionality look-up: to account for errors in the automatic extraction of the mutation directionality (i.e. wild type residue with respect to mutant residue), we examined whether the mutant residue could be matched to the corresponding sequence position. (4) Pro-peptides and mature protein mutation mapping: to handle alternative residue counting when signal peptide cleavage is considered we analyzed positional wild type residue mapping for cases of proteins with N-terminal signal peptide sequences. (5) Methionine start site counting: we carried out mutation mapping taking into account as well as neglecting the N-terminal methionine.

## Competing interests

The authors declare that they have no competing interests.

## Authors' contributions

AV conceived the idea. AV, JMGI and MK planned the analysis. MK, JMGI and CR generated the datasets. MK and JMGI performed the analysis. AV and MK wrote the first draft and MK and JMGI the final version. All authors read and approved the manuscript.

## Supplementary Material

Additional file 1 Click here for file

Additional file 2 Click here for file

Additional file 3 Click here for file

Additional file 4 Click here for file
