# Supplementary material for: Extraction of human kinase mutations from literature, databases and genotyping studies
Source: BMC Bioinformatics. 2009 Aug 27;10(Suppl 8):S1. doi: 10.1186/1471-2105-10-S8-S1 (PMC2745582; doi:10.1186/1471-2105-10-S8-S1)
Supplement: Additional file 1 [file 1471-2105-10-S8-S1-S1.pdf]

Literature mining approaches for mutation extraction

Despite difficulties in extracting more complex language expressions referring to mutation mentions, regularities in describing mutations based on existing nomenclature conventions, promoted the implementation of automated information extraction and text mining systems for the identification of mutations in the literature [1–12]. Text mining assisted literature curation with minimal human processing, has been successfully applied to build a database of literature-derived mutations for serine proteases (CoagMDB) by applying regular expressions to identify amino acids and numeric elements.
Regular expressions additionally have been explored by the MEMA method to retrieve mutation mentions together with HUGO gene names. This system has been cross-validated against information contained in OMIM, and assessed on a set of 100 abstracts known to contain mutation mentions, obtaining a precision of 93.4% and an estimated recall of 35.2%. To provide literature-based pairs of genes/proteins and mutations, MEMA uses information on co-occurrence within sentences, as well as proximity of mentions in abstracts [1]. Similarly MuteXt also uses regular expressions for detecting mutations and co-mention proximity scores to disambiguate between multiple protein terms that might be associated to a given mutation [9]. This system specifically focus on extracting protein point mutations for the GPCR and NR superfamilies from both abstracts and full text articles, integrating a sequence validation check using SwissProt records.
Yip and colleagues carried out a more extensive analysis on the importance and characteristics of positional validation of mutations mentions against information contained in the Swissprot database [10]. They extracted protein mutations from the literature using four regular expressions, evaluating in detail the performance of each them. For the positional validation of the extracted mutations, they accepted one position sequence shift to account for the initiator methionine cleavage effect in the resulting numbering. Additional sequence variation resulting from posttranslational cleavage and alternative splicing was also analyzed in depth, showing that this sequence correction step could account for up to 20% of the validated cases.
The Mutation GraB (Graph Bigram) approach proposed by Lee et al, has been applied to identify point mutations from articles for proteins belonging to the GPCR, tyrosine kinases and ion channel protein families [2]. Linking of the extracted mutations to proteins and organisms is based on positional and frequency information of all terms in a given full text article. The extracted mutation-protein pairs were validated against information contained in the SwissProt database. Disambiguation in cases where several proteins could potentially be associated to a single mutation mention is addressed by this system through a scoring each pair according to a graph shorted distance search in combination with word bigram analysis. A Natural Language Processing (NLP) oriented approach was followed by Mutation Miner, extracting associations between mutations and proteins from sentences where both entities are co-mentioned, while the relations between proteins and the corresponding organism source is extracted through template-based detection of certain noun phrase patterns [3]. A range of other mutation and genomic variant mention detection systems have been published recently, most of them showed a considerable high performance in terms of precision, with greater variability when comparing the obtained recall. Among these strategies are

MuGeX (Mutation Gene eXtractor), for extracting mutation-gene pairs from Medline abstracts given a disease query (i.e. Alzheimer) [6], where machine learning techniques were applied to disambiguate between mutations at the level of protein and DNA sequences. To enable management and visualization of information derived from sequence and structural data with automatically extracted mutations detected from full text articles by NLP methods the mSTRAP (Mutation extraction and STRucture Annotation Pipeline) system makes use of a specifically designed ontology [11]. Machine learning (ML) techniques are increasingly being used to identify mentions of biological entities in the literature, and have been used in case of the VTag system to detect mentions of acquired sequence variations (point mutations, translocations, deletions) in text. VTag relies on CRFs trained on a collection of 345 abstracts manually labeled by domain experts [8]. At the level of document retrieval, another ML method, an maximum entropy classifier had been used to identify abstracts relevant for annotating genomic variation information for the CDKN2A (p16) gene [7].

Another sequence variation entity recognition system had been integrated into the OSIRISv1.2 application, focusing specifically on the detection of human gene variations corresponding to SNPs [13]. A previous version of this system covered the extraction of sequence variations located in the gene and its vicinity (SNPs, insertion/deletion polymorpshims, microsatellite and named variations /Alu sequences), both nucleotidic and amino acid alleles [14].

Although previously published mutation extraction strategies demonstrated the capabilities of automated text mining for detecting sequence variations from the literature, also some practical limitations became evident. Some of these systems show only limited online access to the obtained results or are not suitable for direct exploitation by manual literature curation due do missing links of mutations to the corresponding protein sequences (i.e. database records), a crucial aspect also for integration of sequence and structural information required for bioinformatics analysis. Even though most of the existing manually curated mutations annotation resources are based on reading full text articles, existing automated systems mainly relied only on (subsets of) PubMed abstracts or a small collection of full text articles. To facilitate the interpretation of the biological implications and phenotypic effects of a given mutation, not only by clinical experts but also by database curators or for designing biochemical experiments (drug design and molecular functional studies) it is crucial to know whether a given mutations has been experimentally generated or consists in a naturally occurring sequence variation. This aspect has generally been neglected by previously developed approaches. Finally only few systems were able to show results based on the combination of heterogeneous data derived from multiple information sources, derived from literature as well as based on information obtained by sequence and protein domain analysis.

References

1. Rebholz-Schuhmann D, Marcel S, Albert S, Tolle R, Casari G, Kirsch H: Automatic extraction of mutations from Medline and cross-validation with OMIM. Nucl. Acids Res. 2004, 32:135–142, [http://dx.doi.org/10.1093/nar/gkh162].

2. Lee LC, Horn F, Cohen FE: Automatic Extraction of Protein Point Mutations Using a Graph Bigram Association. PLoS Comput Biol 2007, 3:e16–e16.

3. Baker CJO, Witte R: Mutation Mining—A Prospector's Tale. Information Systems Frontiers (ISF) 2006, 8:47–57.

4. Witte R, Baker CJO: Towards A Systematic Evaluation of Protein Mutation Extraction Systems. Journal of Bioinformatics and Computational Biology (JBCB) 2007, 5(6):1339–1359. [http://rene- witte.net/evaluation- of- mutation- extraction- systems].
5. Caporaso JG, Baumgartner WA, Randolph DA, Cohen KB, Hunter L: MutationFinder: a High-performance System for Extracting Point Mutation Mentions from text. Bioinformatics 2007, 23:1862–1865.
6. Erdogmus M, Sezerman OU: Application of Automatic Mutation-gene pair Extraction to Diseases. J Bioinform Comput Biol 2007, 5:1261–1275.
7. McDonald R, Scott Winters R, Ankuda CK, Murphy JA, Rogers AE, Pereira F, Greenblatt MS, White PS: An
Automated Procedure to Identify Biomedical Articles that Contain Cancer-associated gene Variants. Hum Mutat 2006, 27:957–964.
8. McDonald RT, Winters RS, Mandel M, Jin Y, White PS, Pereira F: An Entity Tagger for Recognizing Acquired Genomic Variations in Cancer Literature. Bioinformatics 2004, 20:3249–3251.
9. Horn F, Lau AL, Cohen FE: Automated Extraction of Mutation data from the Literature: Application of MuteXt to G Protein-coupled Receptors and Nuclear Hormone Receptors. Bioinformatics 2004, 20:557–568.
10. Yip YL, Lachenal N, Pillet V, Veuthey AL: Retrieving Mutation-specific Information for Human Proteins in UniProt/Swiss-Prot Knowledgebase. J Bioinform Comput Biol 2007, 5:1215–1231.
11. Kanagasabai R, Choo KH, Ranganathan S, Baker CJO: A Workflow for Mutation Extraction and Structure Annotation. J Bioinform Comput Biol 2007, 5:1319–1337.
12. Yip YL, Famiglietti M, Gos A, Duek PD, David FPA, Gateau A, Bairoch A: Annotating Single Amino acid Polymorphisms in the UniProt/Swiss-Prot Knowledgebase. Hum Mutat 2008, 29:361–366.

13. Furlong LI, Dach H, Hofmann-Apitius M, Sanz F: OSIRISv1.2: a Named Entity Recognition System for Sequence Variants of Genes in Biomedical Literature. BMC Bioinformatics 2008, 9:84–84.
14. Bonis J, Furlong LI, Sanz F: OSIRIS: a tool for Retrieving Literature About Sequence Variants. Bioinformatics 2006, 22:2567–2569.
15. Caporaso JG, Baumgartner WA, Randolph DA, Cohen KB, Hunter L: Rapid Pattern Development for Concept Recognition Systems: Application to Point Mutations. J Bioinform Comput Biol 2007, 5:1233–1259.
