# Supplementary figures and images for: Extraction of human kinase mutations from literature, databases and genotyping studies
Source: BMC Bioinformatics. 2009 Aug 27;10(Suppl 8):S1. doi: 10.1186/1471-2105-10-S8-S1 (PMC2745582; doi:10.1186/1471-2105-10-S8-S1)

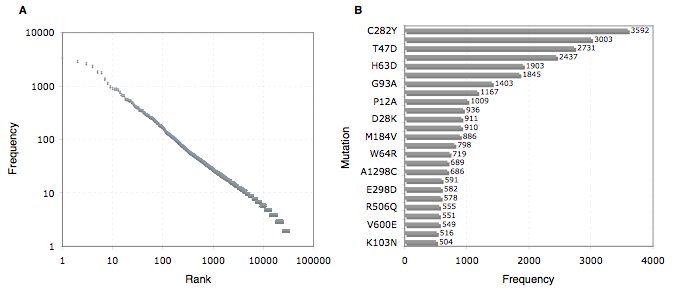

Supplement: Additional file 2 [file 1471-2105-10-S8-S1-S2.png]

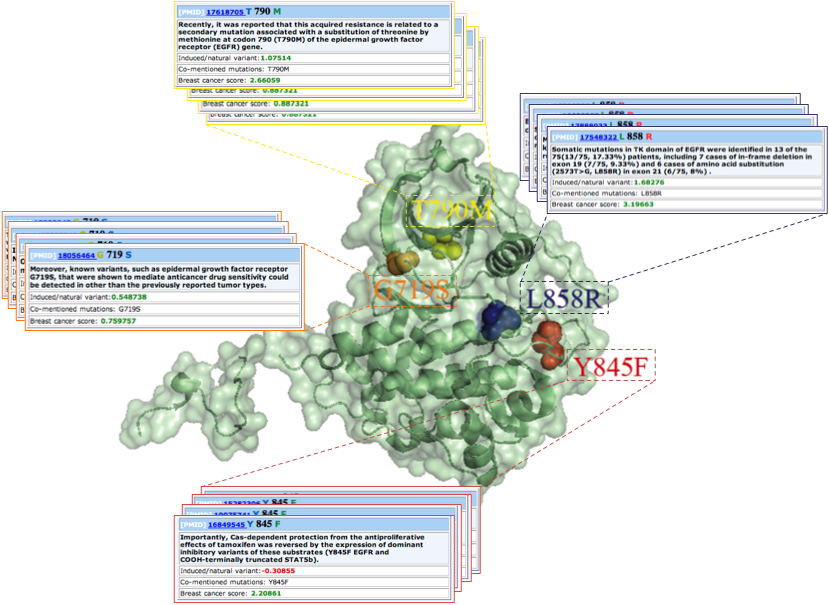

Supplement: Additional file 3 [file 1471-2105-10-S8-S1-S3.png]
